# DIFFERENCES IN COMMUNITY AND PROVIDER STAKEHOLDERS' PERCEPTIONS ON BRAIN HEALTH SERVICE GAPS AND SCREENING NEEDS

**DOI:** 10.1093/geroni/igac059.1851

**Published:** 2022-12-20

**Authors:** Annie Rhodes, Lana Sargent, Faika Zanjani

**Affiliations:** Virginia Commonwealth University, Richmond, Virginia; Virginia Commonwealth University, Richmond, Virginia; Virginia Commonwealth University, Richmond, Virginia

## Abstract

**Introduction:**

Alzheimer's Disease and Related Dementias (ADRD) management and prevention is a priority for providers and community members. Aligning perceptions regarding resources and screening supports person-centered care while addressing the increased dementia burden attributed to systemic inequity and health disparities. This project aimed to identify needs in lower-income diverse populations.Research Question: What are the differences in perceived service gaps and screening preferences for brain health and ADRD?

**Methods:**

A convenience sample of 15 providers and 20 community stakeholders (55+) completed a 2021 survey about Richmond's service gaps and screening needs for brain health and ADRD.

**Results:**

40% of providers were ADRD focused, 65% of community members reported a memory concern. Overall, providers reported fewer service gaps: Administration on Aging Service Gaps 46.7%/75%, Caregiving gaps 80%/85%, Specialty Health gaps: 53.3%/80%. Clinical ADRD gaps 73.3%/80%, Memory Case Management gaps 86.7%/90%, Memory Social Services gaps 33.3%/90%, Memory Screening gaps 53.3%/85%. Provider and Community stakeholders were more aligned regarding screenings that should be included in a brain health/ADRD management program: Advance Care Planning screening: 80%/80%, Caregiving Status screening: 93.3%/90%, Memory Loss screening: Lifestyle Risk screening: 100%/95%, Cognitive-Comorbidity Risks screening: 100%/75%, Psychosocial Risks screening:100%/100%, Depression screening 100%/100%, Clinical Health screening 73.3%/90%, Substance Use Disorder screening 86%/75%, Sleep Problem screening 93%/75%

**Conclusion:**

Although there were areas in which both providers and community members aligned, results show that providers underestimate brain health/ADRD service gaps experienced by the community. Both groups are amenable to comprehensive screening for services. However, providers are interested in more expansive ADRD screeners.

